# Summary of evidence on prevention and management of bladder dysfunction in patients after radical hysterectomy

**DOI:** 10.1002/nop2.2240

**Published:** 2024-07-11

**Authors:** Chao Zeng, Yuanyuan Mi, Fulan Wang, Qinghua Zhao, Mingzhao Xiao, Feng Xiao, Yan Hu, Lin Wang, Fang He

**Affiliations:** ^1^ Department of Gynecology The First Affiliated Hospital of Chongqing Medical University Chongqing China; ^2^ Department of Nursing The First Affiliated Hospital of Chongqing Medical University Chongqing China; ^3^ Department of Critical Care Medicine, Union Hospital, Tongji Medical College Huazhong University of Science and Technology Wuhan China; ^4^ Department of Urology Surgery The First Affiliated Hospital of Chongqing Medical University Chongqing China

**Keywords:** bladder dysfunction, cervical cancer, evidence‐based summary, management, prevention, radical hysterectomy

## Abstract

**Aim:**

To retrieve, analyse and summarize the relevant evidence on the prevention and management of bladder dysfunction in patients with cervical ancer after radical hysterectomy.

**Design:**

Overview of systematic reviews.

**Methods:**

11 databases were searched for relevant studies from top to bottom according to the ‘6S’ model of evidence‐based resources. Two independent reviewers selected the articles, extracted the data and appraised the quality of the included reviews based on different types of evaluation tools.

**Results:**

A total of 13 studies were identified, including four clinical consultants, four guidelines, four systematic reviews and one randomized controlled trial. 29 best evidence were summarized from five aspects, including definition, risk factors, assessment, prevention and management.

## 1 INTRODUCTION

Cervical cancer is the fourth most common cancer in women worldwide, with an estimated 604,000 new cases and 342,000 deaths reported in 2020 (Sung et al., 2021). In the United States, it is the most common gynaecological surgery, second to caesarean section (Zhao et al., 2021). In China as well, the incidence of cervical cancer is high, with an estimated 131,500 new cases diagnosed every year, accounting for 28.8% of the total cases (Buskwofie et al., 2020). Treatment for cervical cancer depends on the severity of the disease. Women with early‐stage cervical cancer (FIGO stages IA2–IIA2) should undergo total pelvic hysterectomy (i.e. removal of the uterus and surrounding tissues and pelvic lymph nodes) or pelvic chemotherapy (lower abdominal chemotherapy plus abdominal radiation) (Bhatla et al., 2019). One of the most painful conditions that can happen after a radical hysterectomy (RH) is bladder dysfunction (i.e. concerning how the bladder stores and releases urine) (Laterza et al., 2015; Plotti et al., 2011). Bladder dysfunction has been reported in 70%–85% of cervical cancer peoples after RH (Wit & Horenblas, 2014). Urinary retention is the main symptom of short‐term bladder dysfunction, while urinary incontinence is the main symptom of long‐term bladder dysfunction after surgery (Axelsen & Petersen, 2006; Zullo et al., 2003). The causes of post‐RH bladder dysfunction include injury to the pelvic autonomic nerves innervating the bladder muscle (detrusor), urethral sphincter and pelvic floor fascia (Aue‐Aungkul et al., 2021). Bladder dysfunction after RH includes urinary retention, urinary incontinence, urinary tract infection, stress urinary tract infection and other lower urinary tract diseases (Aue‐Aungkul et al., 2021). Bladder dysfunction increases the likelihood of urinary tract infection, hospitalization or admission, patient dissatisfaction and the need for intermittent self‐catheterization (Manchana et al., 2010), which has a negative impact on the patient's quality of life (QoL), which can often cause embarrassment or even social isolation (Zhou et al., 2016). Therefore, it is particularly important to prevent and manage bladder dysfunction in patients after RH.

In recent years, scholars have attempted to prevent and manage bladder dysfunction after RH, which includes pharmacological interventions, early postoperative bladder training, low‐frequency electrical stimulation, intermittent catheterization, acupuncture therapy and indwelling catheterization. Most of these methods have been proven to be effective and can promote the bladder function rehabilitation of patients to a certain extent (Jackson et al., 2019; Li et al., 2021; Shen et al., 2020). Furthermore, intermittent catheterization has become the management method of choice for bladder dysfunction, as also recognized by the International Association for Urinary Control (Engberg et al., 2020). However, some researchers recommend the use of caution with the clinical application of these measures.

Evidence‐based practice has been applied to nightingales in 19th‐century medicine in the 1970s to breastfeeding in the late 90s (Mackey & Bassendowski, 2017). It is based on the premise that patient care should be evidence‐based. Nurses use and synthesize the best evidence to guide clinical practice and make decisions (Balakas & Smith, 2016; Mackey & Bassendowski, 2017). To apply clinical interventions more scientifically and normatively, some scholars use evidence‐based summary of evidence on bladder management after cervical cancer surgery. Owing to the small number and low quality of the included literature in this review, the summarized evidence warrants further verification. In addition, the evidence focused on the management of bladder dysfunction and prevention of bladder dysfunction has rarely been mentioned in the relevant literature.

The purpose of this review is to (1) provide references for clinical practice in the prevention and management of bladder dysfunction for medical staff; (2) reduce the incidence of bladder dysfunction after radical surgery; (3) improve the quality of life of patients with cervical cancer after RH. To this end, we collected and evaluated the evidence obtained through evidence‐based nursing methods and summarized the findings.

## 2 METHODS

### 2.1 Establishment of the problem

We developed evidence‐based questions using the Joanna Briggs Institute (JBI) Center for Evidence‐Based Medicine PIPOST question development model. P (population): the people who use the evidence are patients with bladder dysfunction after RH; I (Intervention): Intervention measures are recommended intervention, including prevention and management of bladder dysfunction; P (professional): Clinical medical staff; O (outcome): Incidence of postoperative urinary retention, Replacement rate of urinary catheter, Time of first catheter removal, Catheter‐Associated Urinary Tract Infections (CAUTI), Quality of life (QoL). S (setting) is the evidence application site, that Mainly refers to clinical departments. T (type of evidence) is the type of evidence resource that includes guidelines, expert consensus, clinical decisions, recommended practices, best practice information booklets, systematic reviews, evidence summaries and randomized controlled trials (RCTs).

### 2.2 Evidence sources and retrieval strategies

We followed a comprehensive search strategy to identify the relevant articles. The databases searched included the British Medical Journal Best Practice, UpToDate, Joanna Briggs Institute, Cochrane Library, the National Institute for Health and Care Excellence, the Scottish Intercollegiate Guidelines Network, the Agency for Healthcare Research and Quality, the Guidelines International Network, PubMed, Embase and the Web of Science. The search terms included cervical cancer; cancer, cervical; uterine cervical neoplasm*; neoplasm, uterine cervical; hysterectom*; radical hysterectomy; colpohysterectom*; urinary bladder, neurogenic; neurogenic urinary bladder; bladder, neurogenic; neurogenic bladder; urinary bladder neurogenic dysfunction; urinary retention; urinary tract infection; systematic review; meta‐analysis*; randomized controlled trial; guidelines; evidence summaries; expert consensus; clinical decisions. The search time limit was set from inception until October 2022.

### 2.3 Study inclusion and exclusion criteria

The study inclusion criteria were as follows: (I) The main study content includes measures related to the prevention and management of bladder dysfunction; (II) the study population is composed of patients with bladder dysfunction after RH; (III) the outcome indicators include the Incidence of postoperative urinary retention, Replacement rate of urinary catheter, Time of first catheter removal; Catheter‐Associated Urinary Tract Infections (CAUTI); (IV) articles published in both English and non‐English languages; (V) the types of articles included were systematic reviews; meta‐analysis; RCTs; guidelines; evidence summaries; expert consensus; clinical decisions.

The study exclusion criteria were as follows: (I) incomplete literature information, or only a brief literature or research plan of introduction, abstract and draft; (II) repeated or translated literature; (III) non‐availability of the full‐text; (IV) documents evaluated as low quality.

### 2.4 Data extraction

Two researchers independently completed the data extraction. First, all searched data were imported into ENDNOTE software and checked for duplicates. Second, for the initial screening, based on the inclusion and exclusion criteria, articles were selected after reading the title and abstract of the paper. Third, the full text was carefully read to select the target article. When selecting the final documents for inclusion, two researchers could mutually discuss to reach a consensus or invite other evidence‐based nursing experts.

### 2.5 Literature quality evaluation

#### 2.5.1 Literature quality evaluation process

The literature quality evaluation was independently completed by two authors. In terms of education, the author attended and completed the JBI Evidence‐Based Nursing Collaborative Center course at Fudan University. In case of any disagreement, the two authors resolved the differences through discussion, and, if no agreement could be reached, an evidence‐based nursing expert and an evidence‐based trainer were consulted.

#### 2.5.2 Literature quality evaluation method

##### Evaluation of guidelines

The best practice, clinical decision‐making and evidence summary with high quality were directly extracted. The guideline was evaluated with reference to the Appraisal of Guidelines for Research and Evaluation Instrument (AGREEII) (Gavriilidis et al., 2020), which was updated in 2012 in the United Kingdom. It includes six dimensions and 23 elements. Four professional experts in the production and procedure of guidelines were invited to evaluate the included guidelines, and each expert read the guideline carefully and scored independently as per the evaluation tool. Each point was scored on a scale of 1–7, where 1 = fully not agreeing and 7 = fully agreeing, and the scores for each dimension were synthesized using scores of all entries in that dimension, followed by normalization of the results. The formula for standardized processing presented as follows:

$$\left(\text{actual score}-\text{lowest possible score}\right)/\left(\text{highest possible score}-\text{lowest possible score}\right)\times 100\%$$

##### Evaluation of systematic reviews

Systematic reviews were evaluated independently through the assessment of multiple systematic reviews (JBI2016) (Shea et al., 2007) by two professionals. The JBI 2016 scale has a total of 11 items, and the evaluation options are ‘yes’, ‘no’, ‘unclear’ and ‘not applicable’. Items rated “yes” = 1 point; unclear or not mentioned = 0.5 points; no = 0 points. The maximum score is 11 points. The overall quality grade is divided into high, medium and low. The score range is 0–4 = low quality, 5–8 = medium quality and 9–11 = high quality.

##### Evaluation of RCTs

RCT reports adopting the corresponding evaluation standard of the Australian JBI Evidence‐Based Health Care Center (Tufanaru et al., 2020) were evaluated independently by two professionals. There were 13 items in total, and the evaluation options were ‘Yes’, ‘No’, ‘Unclear’ and ‘Not applicable’.

### 2.6 Evidence summaries, classifications and recommendation levels

Three clinical nursing staff with work experience in the department of gynecology at least 10 years attended to extract the evidence according to the research question, followed by a review of the evidence. Based on the FAME principle (‘feasibility, suitability, clinical significance and effectiveness’ of the evidence) and after combining the actual needs of patients from clinics, the evidence was summarized. The evidence was listed based on five aspects: definition, risk factors, assessment, prevention and management after discussion with nursing experts. The 2014 version of the JBI Evidence Pre‐grading and Evidence Recommendation Grade System was adopted to assess and classify the included evidence (Munn et al., 2014). Following the JBI evidence recommendation strength grading principle, we clarified the recommendation level of the evidence. The system divide the evidence level into five levels, and the recommendation opinions were classified as A = strongly recommended and B = weak recommendation.

## 3 RESULTS

### 3.1 Search studies

**FIGURE 1:**
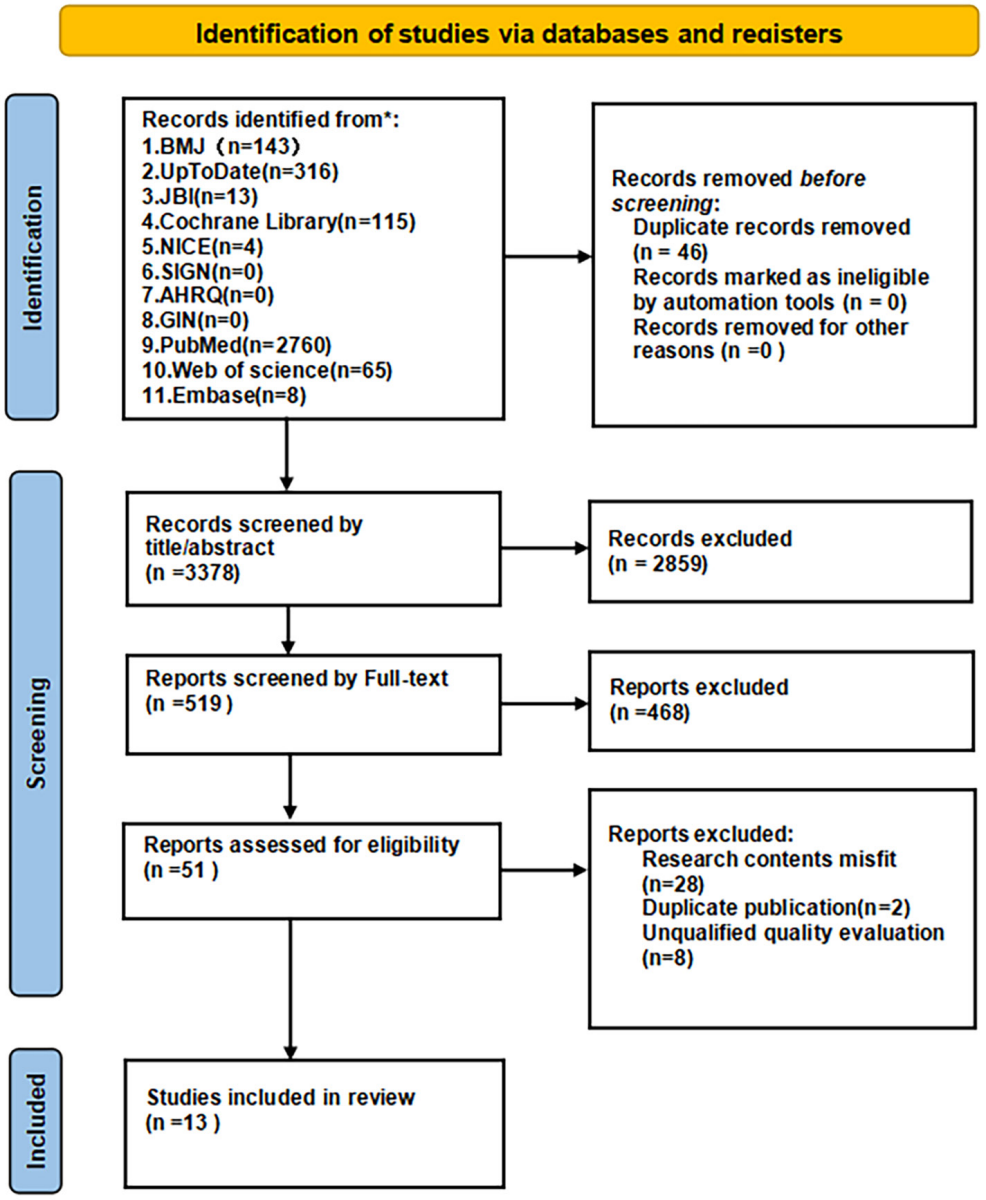
Preferred Reporting Items for systematic Reviews and Meta Analyses (PRISMA) flowchart 2020 describing the study selection process. Flow diagram depicting the process of study searches and screening, which involved searching databases, checking for duplicates, reading the full text and screening based on the inclusion criteria.

The retrieval flow chart depicted in Figure 1. A total of 3424 studies were retrieved and imported into the ENDNOTE software. After screening the titles and abstracts, 2859 papers were excluded. A total of 519 studies were screened by full text based on the inclusion and exclusion criteria, and 468 studies were excluded. A total of 51 studies were assessed for eligibility, and 38 studies were excluded because they were deemed unsuitable for quality assessment due to inconsistencies in the study contents and duplicate publications. Finally, 13 studies were identified in the review, which included 4 clinical consultants, 4 guidelines, 4 systematic reviews and 1 RCT. See Table 1 for general information about the included studies.

**TABLE 1:** General information included in the studies.

Inclusion study	Years	Evidence source	Evidence theme	Evidence type
William J	2022	Up to date	Radical hysterectomy	Clinical Consultant
Linda R	2022	Up to date	Overview of the management of cervical cancer survivors	Clinical Consultant
Anthony J	2021	Up to date	Placement and treatment of adult urinary catheter	Clinical Consultant
Anthony J	2021	Up to date	Complications of urinary catheter and preventive strategies	Clinical Consultant
Ginsberg D	2021	AUA Guideline	Guideline on Adult Neurogenic Lower Urinary Tract Dysfunction: Diagnosis and Evaluation	Guideline
Kavanagh A	2019	Cochrane	Association guideline for the diagnosis, management and surveillance of neurogenic lower urinary tract dysfunction	Guideline
Gould CV	2010	PubMed	Guideline for Prevention of Catheter‐Associated Urinary Tract Infections	Guideline
Game X	2020	PubMed	Guideline for Intermittent catheterization	Guideline
Aue‐aungkul	2021	Cochrane	Postoperative interventions for preventing bladder dysfunction	Systematic review
Sirisreetreerux	2021	Web of science	Medical and non‐medical interventions for post‐operative urinary retention prevention	Systematic review
Zheng	2021	PubMed	Effectiveness of Acupuncture on Urinary Retention	Systematic review
Zhao Q	2021	PubMed	Efficacy and safety of acupuncture for urinary retention after hysterectomy	Systematic review
Zong J	2021	PubMed	Kegel Pelvic Floor Muscle Exercise Combined with Clean Intermittent Self‐catheterization on urinary retention	Randomized controlled

*Note*: The documents were listed and displayed by author name, year of publication, document subject and document type.

### 3.2 Evaluation of publication quality

#### 3.2.1 Quality evaluation of guidelines

There were 4 guidelines (Gamé et al., 2020; Ginsberg et al., 2021; Gould et al., 2010; Kavanagh et al., 2019) included in this study. In the guideline of Ginsberg et al., 2021, the percentage of scores in two dimensions included applicability and independence <30% and the other dimension >60%. In the guideline of Kavanagh et al., 2019, each dimension was >30% and the dimension of rigour reached close to 60%. In the guideline of Gould et al., 2010 and Gamé et al., 2020, each dimension was >60%. The percentage of standardization in each field and the average score of the two comprehensive evaluations were depicted in Table 2.

**TABLE 2:** Quality evaluation of the guideline.

Included literature	Percentage of standardized scores in each dimension (%)					
	Scope and purpose	Participants	The rigour of the guideline development	Clarity of the guideline presentation	Applicability of the guidelines	Independence of the guide
Ginsberg D	91.66%	79.16%	67.20%	70.83%	19.44%	22.91%
Kavanagh A	90.27%	66.16%	59.52%	56.25%	43.05%	79.16%
Gould CV	94.44%	87.50%	80.35%	63.41%	81.94%	93.75%
Game X	95.83%	84.38%	73.20%	83.33%	69.44%	93.75%

*Note*: The quality of the included guidelines was evaluated in six dimensions by four professionals; the higher the proportion of each dimension, the better the guideline quality.

#### 3.2.2 Quality evaluation of systematic reviews

Among the four systematic reviews included in this study, all study items of three articles (i.e. Aue‐Aungkul et al., 2021; Zhao et al., 2021; Zheng et al., 2021) were evaluated as ‘yes’, with complete research design and high overall quality, and hence approved for inclusion. One of these studies (i.e. Sirisreetreerux et al., 2021), had all entries ‘yes’ except for 11 (‘Whether appropriate suggestions were made for the specific direction of further research?’). The literature was included after an overall independent valuation by two researchers. The quality results of the systematic reviews were shown in Table 3.

**TABLE 3:** Quality evaluation of the Systematic review.

Included literature	1	2	3	4	5	6	7	8	9	10	11	Total points
Aue‐Aungkul	1	1	1	1	1	1	1	1	1	1	1	11
Sirisreetreerux	1	1	1	1	1	1	1	1	1	1	0	10
Cheng	1	1	1	1	1	1	1	1	1	1	1	11
Qinyu Zhao	1	1	1	1	1	1	1	1	1	1	1	11

*Note*: The included literature was evaluated through the following 11 dimensions by two professionals, and the points were assigned as follows: 0–4 points = low quality, 5–8 points = medium quality, and 9–11 points = high quality. (1) Whether the evidence‐based issues raised were clear; (2) Whether the inclusion criteria of the literature were appropriate; (3) Whether the retrieval strategy adopted was appropriate; (4) Whether the source of the research paper was appropriate; (5) Whether the document quality evaluation standards adopted were appropriate; (6) Whether two or more evaluators independently completed the literature quality evaluation; (7) Whether certain measures were taken to reduce errors when extracting the data; (8) Was the method of comprehensive/combined research appropriate; (9) Were the possible publication biases assessed; (10) Were the recommendations made on policies and/or practices supported by reported data; (11) Whether appropriate suggestions were made for a specific direction of further research.

#### 3.2.3 Quality evaluation of RCT

The study included 1 RCT (Zong et al., 2022), which was sourced from PubMed. It showed all items ‘yes’, except for items 4 (‘blind study of participants’), 5 (‘blind study of interveners’) and 6 (‘Outcome evaluators blindness’) being ‘not applicable’. The study design was relatively complete and the overall quality was relatively high, thus it was approved for inclusion.

### 3.3 Evidence description and summary

#### 3.3.1 Synthesis of evidence

29 best pieces of evidence were summarized based on five aspects: definition, risk factors, evaluation, prevention and management. Of these 29 pieces of evidence, 4 at level 1, 24 at level 5 and 1 at level 3. There were 18 strongly recommended items and 11weakly recommended items. See Table 4 for the specific information about the summarized evidence.

**TABLE 4:** Summary of the best evidence for the prevention and management of bladder dysfunction.

Dimensionality	Contents of evidence	Evidence level	Recommended level
Definition	1. Bladder dysfunction is a common complication following radical hysterectomy, caused by the damage to pelvic autonomic nerves that innervate the muscles of the bladder, urethral sphincter, and pelvic floor fasciae. It includes various functional disorders of the lower urinary tract, such as urinary retention, voiding difficulty, urinary hesitancy, urinary tract infection, and urinary stress incontinence. (Anthony, 2021a, 2021b; Linda, 2022; William, 2022)	Level 5	A
Risk factors	2. Radical hysterectomy; Operation time exceeds 2 hours; The indwelling time of urinary tube exceeds 2 weeks; Urinary tract infection; Insufficient pelvic floor muscle exercise; Increased resistance of bladder outflow tract; Previous history of urinary retention (Anthony, 2021a, 2021b; Linda, 2022; William, 2022)	Level 5	B
Assessment	3. Evaluation of medical history, collection and analysis of detailed medical history of the patient's urinary system, intestinal tract, sexual system and nervous system (Ginsberg et al., 2021; Kavanagh et al., 2019)	Level 5	B
	4. At initial evaluation, patients should undergo a detailed history, physical exam, and urinalysis (Ginsberg et al., 2021; Kavanagh et al., 2019)	Level 5	B
	5. Physical examination: palpate the patient's lower abdomen, perineum, vagina or rectum, and evaluate the patient's perineum, saddle area sensation and reflex (Ginsberg et al., 2021; Kavanagh et al., 2019)	Level 5	B
	6. Laboratory examination/imaging examination to evaluate the patient's routine urine test, urine culture, urinary ultrasound and renal function test (Ginsberg et al., 2021; Kavanagh et al., 2019)	Level 5	B
	7. Urodynamics, especially VideoUDS is the gold standard for patients(Kavanagh et al., 2019)	Level 5	B
Prevention	8. It is recommended to consider nerve‐sparing radical hysterectomy according to the patient's condition (Aue‐Aungkul et al., 2021)	Level 3	B
	9. It is recommended to use drugs to improve the detrusor muscle, shrink and reduce urethral resistance to prevent bladder dysfunction, such as Cholinergic drugs, alpha‐adrenergic antagonist (Aue‐Aungkul et al., 2021; Sirisreetreerux et al., 2021)	Level 1	B
	10. It is recommended use acupuncture for urinary retention after hysterectomy (Zhao et al., 2021; Zheng et al., 2021)	Level 1	B
	11. Recommend patients to get out of bed and early ambulation according to their own conditions after surgery (Sirisreetreerux et al., 2021)	Level 1	A
	12. Recommend patients perform Kegel pelvic floor muscle exercise 3 days before surgery, and diastolic and contractile exercises of the vagina, urethra and anal sphincter were performed on the 3 days after surgery while.lying in bed; supining with legs flexed apart. When inhaling, the perineum and anus were contracted forcibly, lasting about 13. seconds, and when exhaling, it was relaxed about 10 s. The above actions were repeated for about 20 min, with an interval of 5–10 s between each time, three times a day (Zong et al., 2022)	Level 1	B
Management	13. When catheters need to be placed, it is recommended that patients and caregivers of medical staff should be fully trained and educated (Anthony, 2021a, 2021b; Gould et al., 2010)	Level 5	A
	14. It recommend evaluating the timing of catheter removal to remove the catheter as early as possible (Anthony, 2021a, 2021b; Gould et al., 2010)	Level 5	A

	15. Keep the drainage tube unobstructed, always keep the collection bag below the bladder level, and do not put the urine bag on the floor (Anthony, 2021a, 2021b; Gould et al., 2010)	Level 5	A
	16. It is recommended to wash the area around the urethra or above the pubis with water or normal saline every day (Anthony, 2021a, 2021b; Gould et al., 2010)	Level 5	A
	17. It is recommended to use standard precautions to prevent infection whenever handling catheters (Anthony, 2021a, 2021b; Gould et al., 2010)	Level 5	A
	18. If it is necessary to take urine sample or urine culture, after cleaning the port with a sterilizer, use a sterile syringe sleeve adapter to take urine from the needle free sampling port (Anthony, 2021a, 2021b; Gould et al., 2010)	Level 5	A
	19. It is recommended to maintain a continuously closed sterile drainage system. It is not recommended to replace the indwelling catheter or drainage bag at regular or fixed intervals, but to replace the catheter and drainage bag according to clinical indications, such as infection, obstruction or when the closed drainage system is damage (Anthony, 2021a, 2021b; Gould et al., 2010)	Level 5	A
	20. Preventive antibiotics are not recommended for unconventional use (Anthony, 2021a, 2021b; Gould et al., 2010)	Level 5	A
	21. Intermittent catheterization is recommended as the first choice to help patients empty their bladder. Long term indwelling catheterization should be the last choice (Gamé et al., 2020)	Level 5	A
	22. Health education is recommended for patients and their families during intermittent catheterization (Gamé et al., 2020)	Level 5	A
	23. It is recommended to evaluate the residual urine, urination and auxiliary examination results of the patient before Intermittent catheterization (Gamé et al., 2020)	Level 5	A
	24. Hydrophilic coated catheter with pipe diameter of 12–14F shall be preferred for intermittent catheterization (Gamé et al., 2020)	Level 5	A
	25. The frequency of intermittent catheterization is determined according to the residual urine volume of the patient. It is usually 4–6 h each time (Gamé et al., 2020)	Level 5	A
	26. Make drinking water plan, drinking water 1500–2000 mL/day, ≤400 mL/time, and avoid drinking water 3 h before falling asleep (Gould et al., 2010; Gamé et al., 2020)	Level 5	A
	27. Use urination diary to record the patient's catheterization information, fluid intake, urinary system symptoms, and evaluate their lower urinary tract function for more than 7 consecutive days (Gamé et al., 2020)	Level 5	B
	28. Regular follow‐up urine routine examination, urinary ultrasound B ultrasound, residual urine volume measurement, renal function and urodynamics examination are taken as basic follow‐up examination items (Gamé et al., 2020)	Level 5	A
29. During intermittent catheterization at home, patients should be trained and supervised and followed up by medical staff (Gould et al., 2010; Gamé et al., 2020)	Level 5	A

*Note*: The best evidence for the prevention and management of bladder dysfunction is summarized. Five aspects of evidence about bladder dysfunction after radical hysterectomy were summarized, including the definition, risk factors, assessment, prevention and management. Evidence level: The evidence level was divided into five levels: level 1 = highest, level 3 = moderate, level 5 = lowest. According to the different research types, the evidence level was divided into five levels; the recommendation level was divided into A‐level and B‐level, with A level—strongly recommended and B level = weakly recommended.

## 4 DISCUSSION

### 4.1 Significance of evidence summary for the prevention and management of bladder dysfunction in patients after RH

RH with pelvic lymphadenectomy is considered a standard surgical treatment by the Federation of Obstetrics and Gynaecology stages IB1‐IIACC (Shen et al., 2020). As the surgical site is inside the pelvic cavity, the ureter and bladder can get easily damaged during the surgery, possibly leading to bladder complications such as urinary retention and urinary tract infection (Yu et al., 2022). These conditions place heavy physical, psychological and economic burdens on patients for a long time, hence measures should be taken to prevent and manage bladder dysfunction after cervical cancer surgery. Therefore, we attempted to summarize the evidence related to this topic. Accordingly, we summarized the five aspects of the prevention and management of bladder dysfunction in patients after RH. We obtained 29pieces of best evidence in this study, which were based on clinical consultants, guidelines, systematic reviews and RCT, and the overall quality of the evidence in this study was relatively high.

### 4.2 Prevention and management of bladder dysfunction in patients after RH

The evidence derived from articles 1–2 emphasizes the importance of the definition and risk factors for bladder dysfunction in patients after RH. Past research has shown that 70%–85% of all cervical cancer peoples face bladder function symptoms after the surgery, which affects their postoperative rehabilitation experience (Li et al., 2019, 2021). However, the time of occurrence and the recovery of bladder dysfunction after RH is not uniform across cases. Some patients can recover 6 months after surgery, while others have a relatively long illness duration, and some of them can even take more than 1 year to recover. Therefore, it is important to recognize and master the knowledge about the risk factors of bladder dysfunction in patients after RH so as to identify preventive intervention as soon as possible (Plotti et al., 2011). The operation time of RH is relatively long, and the scope of operation is relatively large, as the tissues near the uterus have to be removed, which can damage the nerves at this location and cause bladder dysfunction (Wit & Horenblas, 2014). The duration of catheter indwelling is also related to bladder dysfunction; the longer the catheter indwelling, the higher the incidence of this disease (Fukuoka et al., 2018; Turnbull et al., 2012). The pelvic floor muscle strength and the resistance of the bladder outlet are also important factors that affect postoperative urinary retention (Stabholz & Sandhu, 2021).

The evidence derived from articles 3‐7  illustrated in specific ways the assessment of bladder dysfunction in patients after RH. The guideline (Kavanagh et al., 2019) for the Diagnosis and Evaluation of Neurogenic Lower Urinary Tract Dysfunction in Adults states that all patients with bladder dysfunction should receive a detailed medical history, physical examination and urine examination during the preliminary evaluation. A comprehensive initial assessment is essential to guide subsequent assessment and management. Residual urine should be checked at the time of diagnosis and regularly to monitor the extent of emptying of the bladder. After the preliminary evaluation, the risk of complications remains unknown, and further laboratory tests are needed for auxiliary diagnoses, such as routine urine tests, urine cultures and urinary ultrasound. Although urodynamic examination is the gold standard for evaluating neurogenic bladder dysfunction, it is time‐consuming, expensive and invasive, which can bring discomfort to patients and involve a high risk of urinary tract infection. Ultrasound is a simple,non‐invasiveand highly accepted diagnostic approach by patients. Therefore, ultrasound remains the most commonly used method to evaluate bladder function of patients undergoing cervical cancer surgery.

The evidence derived from articles 8‐12 mainly describes the prevention measures of bladder dysfunction in patients following RH. The preventive measures of bladder dysfunction mainly include pharmacological interventions and non‐pharmacological interventions. Researches have indicated that the main factor affecting bladder dysfunction after a hysterectomy is malfunctioning bladder detrusor (Laterza et al., 2015; Plotti et al., 2011). Parasympathetic pulses mediated by the neurotransmitter acetylcholine can stimulate the contraction of the detrusor muscle of the bladder. The pharmacological intervention mainly involves improving the contraction of the detrusor muscle and reducing urethral resistance, which may have a preventive effect on postoperative bladder dysfunction. Recently, some system reviews (Aue‐Aungkul et al., 2021; Jackson et al., 2019; Sirisreetreerux et al., 2021) explored the usefulness and safety of pharmacological interventions such as antispasmodic agents, non‐steroidal anti‐inflammatory drugs suppository, alpha‐adrenergic antagonist (Ghuman et al., 2018), cholinergic drug and opioid antagonist agents in preventing urinary retention. Past research (Aue‐Aungkul et al., 2021) has indicated that bethanechol, a type of cholinergic preparation, may reduce the chance of bladder dysfunction by lowering the volume of post‐void residual assessed 1 month after the surgery and may show better urodynamic assessment results. However, the certainty of this evidence is relatively low, warranting further research to confirm this outcome. A network meta‐analysis (Sirisreetreerux et al., 2021) revealed that opioid antagonist agents and alpha‐adrenergic antagonists can significantly reduce the incidence of postoperative urinary retention, with no difference in adverse events. In addition, regarding the risk–benefit analysis of the medical treatment, alpha‐adrenergic antagonists show the highest probability of net benefit at the acceptable threshold of a side effect of 15%, followed by opioid antagonist agents and cholinergic drugs (Sirisreetreerux et al., 2021). Non‐pharmacological intervention mainly includes early mobilization and acupuncture therapy. Early mobilization refers to patients having no bed rest and being mobilized to the toilet (Sirisreetreerux et al., 2021). Numerous recent researches have demonstrated that acupuncture regulates neurotransmitters and can objectively improve urodynamics. In addition, to clarify the curative effect of acupuncture on urinary retention, some meta‐analyses have indicated that acupuncture can be applied as a safe and effective treatment approach for women presenting with retention after hysterectomy based on the analyses of the included RCTs (Zhao et al., 2021; Zheng et al., 2021). The frequently used acupuncture points include ciliao (BL32), sanyinjiao (SP6), Zhongji (RN3) and Guanyuan (RN4) (Zheng et al., 2021). However, the acup‐session and checkpoint vary across studies, and different intervention times may have led to different outcomes, which requires further research and screening of fixed and effective treatment time to establish a fixed acupuncture treatment plan for better implementation. Bladder training is an important form of behaviour therapy that can be effective in treating bladder dysfunction following RH as it targets the detrusor muscle and promotes normal bladder filling and emptying (Aue‐Aungkul et al., 2021). Kegel exercise is one way to exercise the bladder function by employing the method of increasing pelvic floor muscle training invented by Kegel, an American obstetrician and gynaecologist, which aims to stimulate the reproductive nerve and increase nerve tension through conscious contraction and relaxation of the vaginal, urethral and anal sphincters, thereby promoting the recovery of bladder functions (Nilsen et al., 2018). Research has shown that pelvic floor muscle exercise can significantly reduce the residual urine volume of patients, improve their urination efficiency and prevent urine reflux (Lucio et al., 2016).

The evidence derived from articles 13‐29 concerns the management of bladder dysfunction in patients after RH. Indwelling catheterization and intermittent catheterization are the main measures for bladder management of patients with cervical cancer after surgery. Early bladder management mainly focuses on indwelling catheterization. However, improper management during indwelling urinary catheters may cause complications such as urinary tract infections, which may affect the bladder rehabilitation of patients (Gould et al., 2010).

Avoiding unwanted catheterization is the most effective measure for reducing catheter complications (Linda, 2022). Therefore, medical staff should regularly evaluate the timing of catheter removal based on the patient's condition and bladder recovery, and remove the catheter in time. In addition, adequate training for patients, medical staff and caregivers can avoid complications related to catheter placement, ensure proper management during catheter retention and ensure timely detection and targeted measurement to avoid or treat complications (Linda, 2022). For the care of urinary catheters, it is recommended to wash the area around the catheter with normal saline and clean it with water every day. During the indwelling of a urinary catheter, the placement of a urinary bag and the smooth drainage of the drainage tube are important measures for avoiding catheter‐related infections (Gould et al., 2010). The use of antibiotics cannot prevent urinary tract infection, rather they can increase the risk of infection from drug‐resistant bacteria. Bladder flushing is only adopted for specific patients, such as postoperative patients or those who need drug treatment or have hematuria. Therefore, it is not recommended that patients routinely use bladder flushing and preventive antibiotic treatment in case of indwelling urinary catheters (Jackson et al., 2019; Zhou et al., 2016). Intermittent catheterization is an alternative method of indwelling catheterization. This approach refers to pulling out of the catheter immediately after the bladder pressure is relieved and conducting regular catheterization to drain urine according to the residual urine and bladder functions of the patient (Aue‐Aungkul et al., 2021). Intermittent catheterization is the golden standard for bladder management of neurogenic bladder dysfunctions. The guideline by Gamé et al. (2020) states that when intermittent catheterization is conducted for patients, health education should also be carried out for the patients and their families to improve their self‐management abilities. The residual urine, urination and bladder function of the patient should be fully evaluated before intermittent catheterization (Li et al., 2021). For the selection of an appropriate intermittent catheter, it is recommended to use hydrophilic‐coated catheters. Research has shown that hydrophilic‐coated catheters are not only conducive to preventing complications but also are more cost‐effective (Xi et al., 2021). During intermittent catheterization, patients should be instructed to carefully implement a drinking water plan and maintain a urination diary, as well as adjust the frequency of intermittent catheterization based on their residual urine volume (Fang et al., 2020). Patients who need intermittent catheterization at home should be guided and supervised by nurses or health care personnel to facilitate their bladder rehabilitation.

## 5 CONCLUSIONS

This study employed evidence‐based nursing to summarize the best evidence for the prevention and management of bladder dysfunction after RH, by mainly including five aspects: the definition, risk factors, evaluation, prevention and management, so as to provide a theoretical basis for clinical medical staff management. Medical personnel can apply evidence to clinical practice and explore the obstacles and promoting factors in the process of evidence application so as to promote the transformation of evidence into clinical practice. Owing to certain cultural differences across regions, a selection bias may have been introduced. It is suggested that, in the process of evidence transformation and application, the clinical manifestations and actual conditions of patients should be collected to provide targeted nursing measures for patients in order to improve their quality of life.

## 6 LIMITATIONS

Some limitations regarding the prevention and management of bladder dysfunction in patients after RH need to be acknowledged. The number of high‐quality randomized controlled studies are limited, therefore, only one randomized controlled study included in this paper. Owing to lacking of the guidelines of bladder management in patients with cervical cancer after RH, we extracted evidence from two guidelines focused on neurogenic bladder function.

## AUTHOR CONTRIBUTIONS

All listed authors certified their contribution. Chao Zeng, Fulan Wang, Yuanyuan Mi: Study design. Chao Zeng, Yuanyuan Mi, Lin Wang, Fang He: searched the database and collected the data. Chao Zeng, Yuanyuan Mi, Lin Wang, Fang He: evaluated the quality of literature. Chao Zeng, Yuanyuan Mi, Lin Wang, Yan Hu: summarized evidence analysis. Chao Zeng, Fulan Wang, Yuanyuan Mi, Qinghua Zhao, Feng Xiao, Mingzhao Xiao: Manuscript writing and revisions for important intellectual content. All authors read and approved the final manuscript.

## FUNDING INFORMATION

The Chongqing Science and Health Joint Medical Research Project (No. 2021MSXM200). Chongqing key specialty construction “Clinical Nursing” quality construction project 0203 [2023] 47‐202336.

## CONFLICT OF INTEREST STATEMENT

The authors declare that they have no conflict of interest.

## ETHICS STATEMENT

The Research Ethics Committee approval was not required.

## Data Availability

The data underlying this article are available in this article and its online supplementary material.
